# Surgery for subclavian arteriovenous fistula with ruptured pseudoaneurysm using total circulatory arrest

**DOI:** 10.1186/1749-8090-8-169

**Published:** 2013-07-05

**Authors:** Tae-Eun Jung, Jin-Tae Kwon, Dong-Hyup Lee, Jang-Hoon Lee, Oog-Jin Shon

**Affiliations:** 1 Department of Thoracic and Cardiovascular Surgery, College of Medicine, Yeungnam University, Daemyeong 5-dong, Nam-gu Daegu 705-717, Korea; 2 Department of Orthopedic Surgery, College of Medicine, Yeungnam University, Daegu, Korea

**Keywords:** Subclavian artery, Arteriovenous fistula, Pseudoaneurysm

## Abstract

Subclavian arteriovenous (AV) fistula is an uncommon disease and rarely occurs secondary to injury. We herein report a case of a ruptured pseudoaneurysm with a subclavian AV fistula caused by clavicle fixation. In cases of a large ruptured pseudoaneurysm with a massive surrounding hematoma, bleeding control and vessel repair is very difficult. For treatment of this case, we decided that median sternotomy and cardiopulmonary bypass with total circulatory arrest would be a good alternative to surgery.

## Background

Subclavian artery injury is uncommon and can reportedly occur from clavicle fracture, gunshot, and other trauma. Likewise, subclavian arteriovenous (AV) fistula, which is also uncommon, can result from trauma or iatrogenesis. Combined pseudoaneurysm and AV fistula of the subclavian artery and vein and its surgical management has rarely been reported in the literature. Rupture of a pseudoaneurysm is especially dangerous to the patient [1-3].

## Case presentation

A 58-year-old Korean female patient had fallen down 4 months previously and suffered a right clavicle shaft fracture. During conservative treatment, the pain continued and open reduction and internal fixation were performed at a local medical center. Preoperative shoulder magnetic resonance imaging showed a pseudoaneurysm around the subclavian artery. However, the pseudoaneurysm was missed. After 1 day, she was transferred to the emergency room because of swelling and pain in the right shoulder, arm and chest areas. She had no past medical history and her vital signs were stable while in the emergency room.

**Figure 1:**
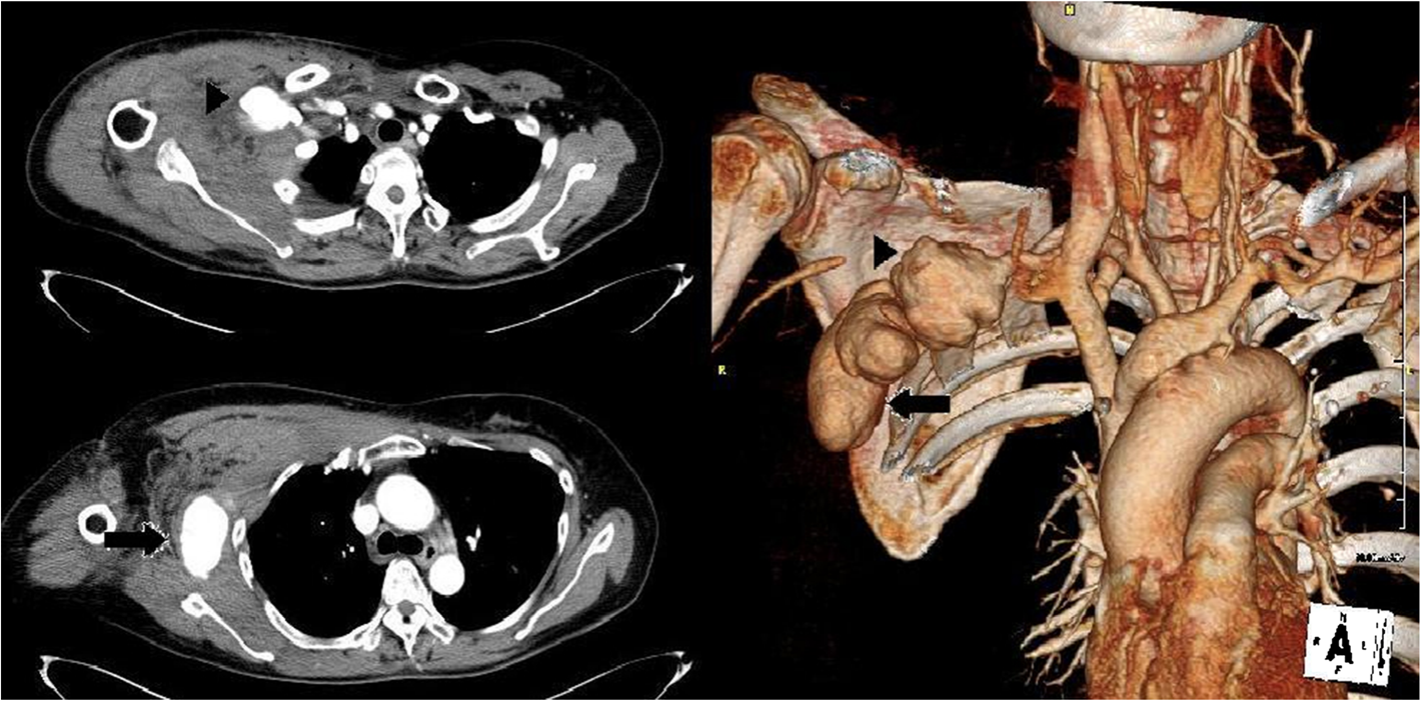
Contrast-enhanced CT scan shows a pseudoaneurysm (arrow head) of the right subclavian artery with a downward protruding pseudoaneurysm (arrow).

On arrival, a tingling sensation in the right arm and pain continued to worsen while the hemoglobin level showed a rapid 4-hour decrease from 8.0 to 4.5 g/dL. We thought that the pseudoaneurysm of the subclavian artery had occurred during the clavicle surgery; thus, three-dimensional computed tomography (3D CT) was performed. Based on the 3D CT findings, we concluded that rupture of the subclavian artery pseudoaneurysm had occurred owing to injury, and emergency surgery was performed (Figure 1). From a supine position, under general anesthesia, the right clavicle was removed to expose the subclavian artery. However, the subclavian artery could not be properly exposed owing to a massive surrounding hematoma and large pseudoaneurysm; in addition, a thrill was felt around the pseudoaneurysm and hematoma. Pseudoaneurysm rupture from the subclavian artery injury was suspected preoperatively; however, a subclavian AV fistula with a pseudoaneurysm was suspected intraoperatively, and the area around the sac was thus carefully isolated. During this isolation process, there was a great deal of blood loss that could not be stopped; therefore, we clamped the subclavian and axillary arteries. The thrill was no longer felt, but the bleeding continued. Subclavian vein exposure was difficult, and we could not identify the venous connection site. During compression of the lesion, we decided to perform a median sternotomy for cardiopulmonary bypass and total circulatory arrest.

**Figure 2:**
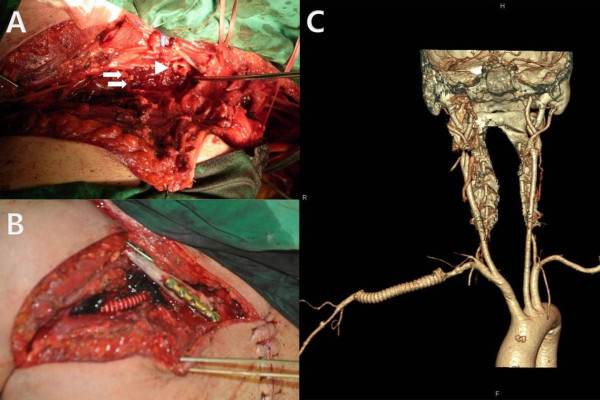
**Open surgical repair. A:** Multiple venous openings (white arrows) were observed in the pseudoaneurysmal sac. **B:** The subclavian artery pseudoaneurysmal sac was removed, and graft interposition was performed on the subclavian and axillary arteries with an 8-mm Ringed Gore-Tex ® Vascular Graft. **C:** Follow-up 3D CT angiogram shows good distal blood flow to the axillary artery through the subclavian artery graft.

The aorta and right atrium were cannulated to perform conventional cardiopulmonary bypass while lowering the body temperature to 20°C, and the aneurysmal sac was then opened. The artery and multiple venous openings were located inside the sac. The pseudoaneurysm was identified through the downward opening, descending along the chest wall. After removal of the hematoma, graft interposition (end-to-side anastomosis with an 8 mm Ringed Gore-Tex® Vascular Graft) was performed on the subclavian and axillary arteries (Figure 2A). Warming began while low-flow circulation was initiated, and the multiple venous openings found internally within the fistula were sutured. The total pump time was 150 min, aortic occlusion time was 69 min, and total circulatory arrest time was 51 min. After closure of the sternum, clavicle reduction and internal fixation were performed using a clavicular compression plate. Ventilator care was started in the intensive care unit. Two days postoperatively, the patient was weaned from the ventilator, and transferred to the general ward. Movement of the right hand was normal and the right radial artery pulse was clearly felt. On postoperative day 18, follow-up CT showed that the graft and distal blood flow from the graft were well maintained (Figure 2B). Swelling of the right shoulder and arm subsided, and the patient was discharged on postoperative day 23 with no further complications. She is currently being monitored.

## Discussion

Complications such as a subclavian artery injury, fistula, and pseudoaneurysm, may be caused by screws used in clavicle fracture reduction with a plate [2,4]. In our case, pseudoaneurysm of the subclavian artery was caused by blunt chest trauma, and tearing of the pseudoaneurysm was suspected to have been caused by drilling and insertion of screws during the clavicle operation. However, a thrill was felt around the pseudoaneurysm and large hematoma. After clavicle removal, we diagnosed a subclavian AV fistula with a ruptured pseudoaneurysm.

Location of a pseudoaneurysm in close proximity to other tissues can result in neurologic symptoms. Postoperative bleeding which formed the hematoma in this case, is believed to have caused numbness of the hand by compressing the nerves and vessels, as described previously [1,5].

Few treatment options for pseudoaneurysm and AV fistulas, such as external ultrasound compression, ultrasound guided thrombin injection, a combination of coils and Onyx, transcatheter coil embolization, and surgical management have been reported. An endovascular prosthesis is currently the primary choice for treatment of subclavian arterio-venous fistulae [6]. Although endovascular treatment for subclavian artery injuries may seem attractive, an open surgical approach should be considered depending on the extent and type of injury and condition of the patient [7]. Use of an open surgical approach allows for the simultaneous treatment of massive bleeding and concomitant distal neurovascular symptoms due to high-pressure hematomas. In our case, emergency surgery was necessary owing to the rapid haemoglobin decrease and sudden worsening of neurologic symptoms.

There are instances in which only an artery graft and vein suture were used after clamping both sides of the artery without sternotomy [8]. We believed that the two possible approaches in our case were an upper partial sternotomy with or without bypass or a femoro-femoral bypass for graft interposition of the subclavian artery. However, bleeding from numerous vein openings in the AV fistula blocked the surgeon’s vision, even after clamping both sides of the artery. Thus, the repair had to be performed after sternotomy, cardiopulmonary bypass, and total circulatory arrest.

## Conclusion

Because continued bleeding from the ruptured aneurysm with AV fistula made the operation difficult, cardiopulmonary bypass with total circulatory arrest was performed, and successful outcome was obtained. We decided that median sternotomy and cardiopulmonary bypass with total circulatory arrest was feasible alternative method.

## Consent

Written informed consent was obtained from the patient for publication of this case report and accompanying images. A copy of the written consent is available for review by the Editor-in-Chief of this journal.

## Abbreviations

AV: Arteriovenous; MRI: Magnetic resonance imaging; CT: Computed tomography.

## Competing interests

The authors declare that they have no competing interests.

## Authors’ contributions

JK, OS, and JL wrote the draft of the manuscript and obtained the written consent. TJ and DL performed the literature review, participated in the manuscript writing, helped with the final writing of the paper, and gave final approval of the manuscript. All authors read and approved the final manuscript.

## References

[B1] Serrano JA Rodríguez P Castro L Serrano P Carpintero P Acute subclavian artery pseudoaneurysm after closed fracture of the clavicle Acta Orthop Belg 2003 69 555 557 14748115

[B2] Mouzopoulos G Morakis E Stamatakos M Tzurbakis M Complications associated with clavicular fracture Orthop Nurs 2009 28 217 224 10.1097/NOR.0b013e3181b579d3 19820620

[B3] Modi MP Shah VR Brachial plexus palsy due to subclavian artery pseudoaneurysm from internal jugular cannulation Indian J Crit Care Med 2007 11 93 95 10.4103/0972-5229.33392

[B4] Fatimi SH Deedar-Ali-Khawaja R Niazi SK Luqman Z Late concomitant pseudoaneurysm and arteriovenous fistula of the subclavian artery: a developing country perspective Vasc Endovascular Surg 2010 44 503 505 10.1177/1538574410372147 20519279

[B5] Ding M Hu J Ni J Lv H Song D Shu C Iatrogenic subclavian arteriovenous fistula: rare complication of plate osteosynthesis of clavicle fracture Orthopedics 2012 35 e287 e289 22310422 10.3928/01477447-20120123-21

[B6] Renger RJ de Bruijn Aarts HC van der Hem LG Endovascular treatment of a pseudoaneurysm of the subclavian artery J Trauma 2003 55 969 971 10.1097/01.TA.0000044632.09973.44 14608176

[B7] Tsutsumi K Saito H Ohkura M Traumatic pseudoaneurysm of the subclavian artery following anterior dislocation of the shoulder: a report of a surgical case Ann Thorac Cardiovasc Surg 2006 12 74 76 16572082

[B8] Chloroyiannis Y Reul GJ Iatrogenic left subclavian artery-to-left brachiocephalic vein fistula Tex Heart Inst J 2004 31 172 174 15212131 PMC427380

